# Importance of Lactobacilli for Human Health

**DOI:** 10.3390/microorganisms12122382

**Published:** 2024-11-21

**Authors:** Piotr B. Heczko, Milena Giemza, Weronika Ponikiewska, Magdalena Strus

**Affiliations:** 1 Department of Bacteriology, Microbial Ecology and Parasitology, Jagiellonian University Medical College, 31-121 Cracow, Poland; magdalena.strus@uj.edu.pl; 2 Prolab Ltd., 30-612 Cracow, Poland; giemza@pro-lab.pl (M.G.); ponikiewska@pro-lab.pl (W.P.)

**Keywords:** Lactobacillus, gut–vaginal microbiota, neonates, innate immunity

## Abstract

As an extraordinarily diverse group of bacteria, lactobacilli are now classified into several genera, many of which still include “Lactobacillus” in their names. Despite their names, this group of lactic acid bacteria comprises microorganisms that are crucial for human health, especially during the early development of the human microbiota and immune system. The interactions between lactobacilli and components of the mucosal immunity lead to its shaping and development, which is possibly considered a prime mover in the advancement of the human immune system. Although much of the evidence backing the pivotal role of lactobacilli in maintaining human health comes from studies on probiotics aiming to elucidate the mechanisms of their functional activities and studies on mucosal immunity in germ-free mice, it is justifiable to extend observations on the properties of the individual probiotic Lactobacillus that are related to health benefits onto other strains sharing common characteristics of the species. In this review, we will discuss the acquisition, presence, and functions of lactobacilli in different human microbiota throughout their whole life, including those arising in the amnion and their interactions with mucosal and immune cells. Examples of immune system modulation by probiotic lactobacilli include their colonic competition for available nutrients, interference with colonization sites, competition for binding sites on gut epithelial cells, bacteriocin production, reduction of colonic pH, and nonspecific stimulation of the immune system.

## 1. Introduction

The *Lactobacillus* group (*sensu lato*), represented by over two hundred species and now classified into dozens of new genera [1], is among the most widely distributed taxa on Earth. It is present in practically all viable environments, in different climate zones, and comes into close interaction with a variety of multicellular host organisms, ranging from plants to primates. This unusual versatility of *Lactobacillus* bacteria is subsequent to its adaptability to various life conditions, achieved thanks to the flexibility of their core metabolic pathways. These pathways include pyruvate metabolism, carbohydrate transport and metabolism, the proteolytic system, amino acid metabolism, and protein synthesis. This metabolic flexibility allows lactobacilli to adjust their protein levels in response to varying environmental conditions, enabling their survival and growth across diverse ecological habitats [2]. Moreover, beyond general stress responses and regulatory mechanisms, specific metabolic pathways can be selectively activated or deactivated, leading to changes in the behavior of the strains [3]. One of the most well-recognized *Lactobacillus* species found in different fermentation products, but also in the human gut microbiome, thanks to its adaptation capabilities and genome plasticity, is *L. (Lactiplantibacillus) plantarum* [4].

Research on probiotics, aimed at uncovering their functional mechanisms, provides much of the evidence emphasizing the critical role of the *Lactobacillus* group in the housekeeping of human health. Although extending these findings to all *Lactobacillus* strains that inhabit human body surfaces or diverse ecological habitats is challenging, it is reasonable to speculate that the beneficial properties observed in specific probiotic strains might be broadly characteristic of the species. Given that probiotic lactobacilli have been historically isolated at random from different habitats within the healthy human microbiome and subsequently characterized, it is likely that many other *Lactobacillus* strains with similar properties exist in the human microbiome but have yet to be identified or studied [5,6].

The human gastrointestinal (GI) tract is home to a diverse and ever-changing array of microorganisms, including the gut microbiota, that play a crucial role in maintaining host homeostasis and regulating metabolic functions. An analysis of various metagenomic studies has revealed a total of 2172 distinct microbial species present in humans, categorized into 12 phyla. Of these, a significant majority—93.5%—are classified under the Proteobacteria, Firmicutes, Actinobacteria, and Bacteroidetes phyla. Within this population, 386 species are known to be strictly anaerobic and are predominantly located in the GI tract [7]. A comprehensive catalog of the gut microbiome’s functional potential has recently been created, identifying a total of 9,879,896 genes from a combination of 249 newly sequenced and 1018 previously published samples [8]. Despite not being the predominant group in the adult human gut microbiome, lactobacilli are essential for the early development of this microbiome.

It has been acknowledged for over a century that the gut microbiome plays a crucial role in health, a concept first explored through Metchnikoff’s groundbreaking research on lactic acid bacteria, particularly *Lactobacillus delbrueckii* ssp. *bulgaricus* [9]. Recent years have brought an increasing amount of data that has led to the appreciation of the infant gut microbiome, and especially of the critical window of its highly dynamic maturation. Recent findings have shed light on the significant influence of the gut microbiome on early life development, with a particular focus on how it affects the maturation of the immune system [8]. While the precise molecular processes involved in neonatal priming in humans remain unclear, recent animal research indicates that the way infants acquire their gut microbiota is shaped by the interplay between bacterial and host factors. Therefore, the timing of bacterial colonization seems to be crucial for the development of the gut microbiome. For instance, *Lactobacillus* species are abundant in the vaginal microbiota during the final stages of pregnancy and are also present in large numbers in human milk. Consequently, at birth, lactobacilli outnumber other genera, such as *Bifidobacterium*, when colonizing the mucosal and skin surfaces of newborns. This initial colonization happens through exposure to the *Lactobacillus*-rich environment of the vagina during delivery and is further supported by breastfeeding, which provides additional lactobacilli from the mother’s milk [10].

Colonization of neonate surfaces with Firmicutes with a predominance of lactobacilli, but also bifidobacteria and coagulase-negative staphylococci, also has an immediate effect on neonate innate immunity via the activation of its different mechanisms, exemplified by its stimulation of the production of tight-junction proteins by gut epithelial cells, resulting in gut impermeability for proteins and other macroparticles, but also bacteria, and thus preventing bacterial translocation from the gut lumen to lymphatics and bloodstream. In preterm neonates, especially those with low and very low birth weights, a lack of gut tightness often leads to neonatal sepsis [11,12,13,14]. Current understanding of gut colonization suggests that the beneficial microbiota, transferred from the adult host to the neonate, is established during early infancy. This early microbial community has lasting effects, extending into adulthood and providing protection against systemic infections. Therefore, both the timing and selection of bacterial strains are critical for the effective artificial colonization of at-risk neonates. The success of this strategy is likely to improve as we identify these, and other, influential factors in humans and validate the outcomes of artificial colonization through clinical trials [15].

This narrative review was written from a medical microbiology standpoint to show the importance of the *Lactobacillus sensu lato* for human organisms by summarizing the data on the interactions between these bacteria and human hosts. Moreover, we tried to minimize information on lactobacilli as probiotics, but it was sometimes impossible to find data in the medical literature for some of the properties of the *Lactobacillus sensu lato* when the investigated strains were regarded as probiotics.

## 2. Early Gut Colonization: Sources of Lactobacilli

There is more and more evidence showing that DNA from *Lactobacillus*, *Bifidobacterium* and other mostly anaerobic bacteria is found in most placenta samples from apparently healthy labors. There are also studies that have found living bacteria in the placenta and amniotic fluid, although their presence in these tissues seems to be related to chorioamnionitis and in utero inflammation [16]. Thus, at the moment, it is safer to accept that bacterial DNA is transferred horizontally from mother to fetus across the placenta under normal physiological conditions. Unmethylated CpG oligodeoxynucleotide motifs within bacterial DNA induce immune responses. Toll-like receptor 9 is activated by specific CpG motifs, which then trigger Th-1-type immune reactions. Even though newborns are generally viewed as immunologically underdeveloped, exposure to bacterial DNA might influence and shape their immune system during fetal development earlier than was once believed [15]. Although there are studies indicating a presence of viable bacteria, but not lactobacilli, in amniotic fluid, evidence is not strong enough to accept that this is a physiological mechanism of maternal–fetal transfer of the bacteria [16,17].

From the moment of birth and in the hours that follow, through interactions with the external environment, such as breathing, breastfeeding, and contact with parents and healthcare providers, the baby’s mouth is exposed to numerous microbes. This exposure initiates the process of permanent colonization of the oral cavity during the postpartum period. Within just twenty-four hours, the first microorganisms, known as pioneer species, begin to establish themselves in the infant’s mouth. At this early stage, the oral cavity is most commonly colonized by Gram-positive cocci, particularly *Streptococcus*, *Staphylococcus*, and *Lactobacillus sensu lato* [18,19].

In fact, the literature on the oral microbiota just after birth is based on studies carried out in the previous century, and what is even worse, using only qualitative cultures [20,21]. Despite the use of rather simple and non-accurate methods, it was possible to demonstrate that bacterial strains obtained from the mother’s vagina and newborn’s mouth were identical [22]. It seems that most of the organisms present in the newborn oral cavity represent only the planktonic, but not the resident, part of the microbiota and that some species which are extremely rarely found in a healthy vagina at birth may represent hospital pathogens. These bacteria do not form the early gut microbiota found in the meconium, which is very similar to the amniotic and vaginal microbiota. Using a modern sequencing approach, He et al. found various *Lactobacillus* operational taxonomic units (OTUs) together with diverse strict anaerobic taxons OTUs, but not those of *Staphylococcus* [23].

After birth, breast milk is crucial for establishing bacterial colonization in the infant’s gut. During the lactation period, the gut undergoes a progressive and sequential colonization by various microbial communities, transitioning from an aerobic, milk-focused microbiome in infancy to a more complex, adult-like microbiome as the child grows [24]. Human milk is a primary source of bacteria for breastfed infants; between 10^5^ and 10^7^ bacteria are ingested daily by a baby consuming about 800 mL/day of milk. Notably, viable *Lactobacillus* and *Bifidobacterium* species are common in the milk of women who have not been treated with antibiotics, with their DNA detectable in most milk samples [25]. Species such as *L. gasseri*, *L. salivarius*, *L. reuteri*, *L. fermentum*, and *B. breve* have been found to be more frequent among the bacteria isolated from human milk compared to others. The origins of these bacteria in breast milk are still debated, with research exploring various potential pathways, including retrograde backflow and the possibility of a neonatal gut-to-mother mammary gland pathway [26,27].

## 3. Further Steps in the Development of the Gut Microbiota

The gut microbiota of vaginally delivered neonates resembles the vaginal microbiome, with high populations of *Lactobacillus sensu lato* and *Prevotella* species [28]; other bacteria, such as the members of the Enterobacteriaceae family—that is, *Escherichia* or *Klebsiella*—and Gram-positive cocci are also present. The occurrence of both of the last groups, which are facultative pathogens, poses the danger of neonatal sepsis, especially in low birth-weight newborns [13]. As individuals age, the diversity of the gut microbiota expands until it stabilizes into a mature adult composition. This adult microbiota is primarily characterized by the dominance of three bacterial phyla and is shaped by factors such as genetics, environment, diet, lifestyle, and gut physiology. By around three years of age, the composition and diversity of a child’s gut microbiota closely resemble those of adults [29]. As noted, *Lactobacillus sensu lato* species found in the gut microbiota, although in relatively small numbers, appear to be crucial for supporting the overall health of the organism.

## 4. Lactobacilli in the Adult Gut Microbiota

The human gut microbiome primarily consists of three bacterial phyla: Bacteroidetes (*Porphyromonas*, *Prevotella*), Firmicutes (*Ruminococcus*, *Clostridium*, and *Eubacterium*), and Actinobacteria (*Bifidobacterium*). While lactobacilli and other facultative anaerobic taxa are present in relatively low quantities within the gut, their regulatory functions in maintaining the gut microbiota are disproportionately significant. This importance has been highlighted by numerous studies focusing on probiotics that contain *Lactobacillus* [30]. Historically, one of the mechanisms of *Lactobacillus*–microbiota interactions has involved the excretion of growth-promoting factors for other microorganisms, which was the first idea for the mechanisms of probiotics proposed by Lilly and Stilwell in 1965 [31]. *Lactobacillus sensu lato* bacteria, after successful early colonization of the gut, become long-life members of the gut microbiota, although their numbers, as well as the number of bifidobacteria, decrease in elderly persons [32]. Again, there are multiple clinical trials with *Lactobacillus*- (and *Bifidobacterium*)-containing probiotics showing that supplementation of the elderly gut microbiota with such bacteria leads to restoration of the gut microbiota composition, and may also prevent the development of the gut carcinoma [33]. Besides genetic aberrations and non-coding RNA, disruptions in the gut microbiota, known as dysbiosis, play a role in the development of colon cancer. Among various interventions, lactic acid bacteria, including *Lactobacillus sensu lato*, have been extensively researched as probiotics for their potential in cancer prevention [6].

After effectively colonizing the gut, lactobacilli engage in direct communication with human epithelial cells. For instance, they trigger transcriptional modifications in the gut epithelium, leading to changes in the expression of numerous genes within just a few hours following oral administration. Most of the human genes involved in down- or up-regulation by lactobacilli code for fatty acid metabolism and cellular growth and development [34]. In relation to regulation of the gut immune response, lactobacilli induce anti-inflammatory activities related to the NF-kappa B complex [35]. Moreover, animal studies also indicate that the presence of *Lactobacillus sensu lato* is associated with the growth of beneficial gut microbiota. The phyla Firmicutes and Bacteroidetes form the core microbiota, accounting for over 90% of the known phylogenetic groups present in the healthy gut of breastfed mice, a composition that closely mirrors the core microbiota in human infants [36]. Lactate produced by microbes strongly induces colonic hyperproliferation in mice, with this effect being most evident after a short-term period of starvation. Additionally, lactate serves as a crucial energy source for small intestinal stem cells, enabling them to maintain their capacity for proliferation and differentiation, as shown in organoid models [37].

Another significant, although not restricted to this group, function of *Lactobacillus* in the human gut microbiota that positively influences health is the production of vitamin K2 (menaquinone). This property was already mentioned 30 years ago in the fundamental work on its synthesis by Bentley and Meganathan [38]. Later, it was revealed that yoghurt-fermenting lactobacilli produce menaquinone-7 [39]. However, contemporary data, although confirming the important role of the gut bacteria in vitamin K2 synthesis, do not link it to particular genera. This remains to be elucidated in the light of the actual bacterial taxonomy.

Alterations in gut microbiota composition are observed in various disease conditions, including cardiovascular disease, cancer, type 2 diabetes mellitus, obesity, colitis, asthma, psychiatric and inflammatory disorders, gut–brain axis disturbances, and numerous immune-related issues. Consequently, modulating the gut microbiota through probiotic bacteria, particularly those from the *Lactobacillus* genus, may help address a variety of health challenges. In fact, a range of degenerative diseases, such as obesity, diabetes, cancer, cardiovascular conditions, liver diseases, and inflammatory bowel disease (IBD), have been shown to be preventable by various *Lactobacillus sensu lato* strains from different species [31].

## 5. The Role of Lactobacilli in Gut Innate and Local Immunity

Initial interactions with pioneer bacteria may play a crucial role in determining subsequent gut maturation, as well as in shaping metabolic and immune responses, ultimately influencing both short- and long-term health outcomes [40].

Newborns enter the world with an underdeveloped and naive acquired immune system, and several aspects of their innate mucosal immunity are not fully matured. As a result, the innate immune components found in human milk play a critical role in supporting the mucosal barrier of the infant’s developing gut. Through breastfeeding, mothers supply their infants with numerous protective agents, some of which have been identified as *Lactobacillus sensu lato* strains in laboratory infection models [41].

As with other aspects of the interactions between lactobacilli and the human organism, most of the information on *Lactobacillus sensu lato* on immunity comes from studies on probiotic lactobacilli in animal models. There are multiple studies showing that probiotic lactobacilli contribute to the host’s well-being by enhancing the balance of intestinal microflora and potentially strengthening the host’s defense mechanisms. The mechanism by which innate immunity is stimulated in mammals and birds relies on the NF-kappa B and p38 MAPK signaling pathways, as demonstrated in mice, cows, pigs, and chickens [42,43,44,45]. It is obvious that this property is not restricted to probiotic strains of lactobacilli, although it is highly individualized among strains.

It is commonly agreed, especially in food science, that intestinal homeostasis—in which the local gut immunity plays an important role—can be re-established by consuming supplements that contain fermented dairy products. These products often contain beneficial microbes such as probiotics, predominantly from the *Lactobacillus sensu lato* genera. Multiple studies carried out on different *Lactobacillus sensu lato* strains commonly stress how crucial they are in modulating the gut immune response within both the innate and mucosal immune systems, such as T, B, and NK lymphocytes [46]. It has been shown that a very broad group of *Lactobacillus* strains representing different species show common characteristics. These include the ability to activate an inflammatory response in macrophages in vitro through mechanisms involving the production of proinflammatory mediators such as cytokines and reactive oxygen species (ROS), as well as their involvement in signaling pathways including NF-kappa B and TLR2 [47,48].

On the other hand, the regulatory functions of lactobacilli in the gut promote the production of anti-inflammatory cytokines, which has also been found [49] to positively impact chronic gastrointestinal issues. The work of probiotic lactobacilli in modulating the inflammatory response from the gut mucosa has also been demonstrated in patients with inflammatory bowel disease (IBD), highlighting their beneficial role in managing such conditions [50]. It should be noted that it is commonly accepted that IBD is linked to alterations in the composition and metabolism of the intestinal microbiota, known as dysbiosis, which interact with host genetic mutations and changing immune responses to gut microbes. However, a definitive causal link between dysbiosis and IBD has not yet been established in humans [51].

There are at least two mechanisms by which *Lactobacillus sensu lato* may ameliorate chronic gut inflammation. Some *Lactobacillus* species possess anti-inflammatory enzymes that decompose ROS, like superoxide dismutase or catalase [52,53]. Observations on the gut microbiota composition in IBD patients, and especially in pediatric patients with new-onset disease, have shown its altered composition, with reductions in the bacteria belonging to the phylum Firmicutes—represented also by *Lactobacillus sensu lato*—supporting this idea [54,55]. On the other hand, increased populations of Proteobacteria are able to induce gut inflammation [56,57]. These data may also indicate an anti-inflammatory role of lactobacilli in vivo related to their anti-oxidative properties. There is also another explanation offered for the beneficial role of *Lactobacillus sensu lato* probiotics in IBD. Animal studies have shown that the NF-kappa B pathway is inhibited by probiotics, which promotes the activation of immune-modulating regulatory T cells and reduces the presence of proinflammatory effector memory T cells in the intestinal mucosa [58].

Immune-related disorders have been connected to a decline in overall bacterial diversity, a reduced presence of *Lactobacillus sensu lato* in a healthy gut microbiota, and a rise in potentially harmful bacteria. Additionally, specific bacteria—not including lactobacilli—have been identified as contributors to allergic reactions in both animal studies and human research [59].

Thus, probiotics, especially those containing *Lactobacillus sensu lato* strains, are proposed to offer potential benefits in preventing and treating certain immune-related diseases through the modulation of the gut microbiota and the regulation of host mucosal immune function. Nonetheless, their effectiveness has been variable, and additional research is needed to confirm these outcomes [60].

## 6. Lactobacilli in the Vaginal Microbiota

Undoubtedly, the most completely documented leading role of the *Lactobacillus sensu lato* bacteria in maintaining homeostasis and protecting a healthy status occurs in the human vagina, where lactobacilli act solely as both pioneering and sentinel organisms, forming and keeping a normal vaginal microbiota composition in the majority of women of childbearing age [61]. Vaginal lactobacilli are a crucial defense against invading pathogens, producing bactericidal compounds and metabolites, as well as modulating the innate immune responses in the vagina [62]. There are suggestions that not all *Lactobacillus* species residing in the vagina are equal in their protective role, and that *L. crispatus* is the most active [63] in this respect. Conversely, a very recent study by Hassan et al. indicates that reduced species diversity, combined with an increased abundance of *L. iners*, *L. gasseri*, and *Gardnerella vaginalis*, could be linked to female infertility [64].

The substantial need to discriminate among the species *Lactobacillus* in the vagina and roles of new taxa and their taxonomic positions should be re-evaluated in the light of the extensive studies on the vaginal microbiota presented by Ravel et al., showing that there are at least several types of microbiota with different predominant species of *Lactobacillus sensu lato* [65]. This study by Ravel et al. documented that not all species of the *Lactobacillus* genera possess the ability to colonize the vaginal epithelium, and that *L. crispatus*, *L. iners*, *L. gasseri*, and *L. jensenii* are listed as the most typical members of this community; however, they have different influences in vaginal health, as shown above. Still, there are very interesting differences in the composition of the *Lactobacillus* populations, related to a mix of cultural, behavioral, genetic, and other as-yet unknown factors [66]. Most probably, these differences are caused by unknown variants in specific receptors in the vaginal epithelium, as well as by various foods, since the gut microbiota serves as a reservoir of vaginal lactobacilli. In fact, the human vaginal microbiota is typically dominated by one or two of the five primary lactobacilli species, and is distinguished by a pH level of below 4.5 [67].

High populations of lactobacilli are constantly supplemented and supported by these strains, which pass from the GI tract over perianal, perineal, and vulvar skin to the vaginal orifice, thanks to unique mechanisms driven by specific genes [68,69]. It is noteworthy that these mechanisms have been utilized in constructing and implementing vaginal probiotic products for oral delivery [70,71]. The abundance of vaginal lactobacilli is also regulated by hormonal interplay, as levels increase in puberty and further during pregnancy [72] and decreases during menopause. Most probably, other factors are also involved in the acquisition of lactobacilli, since it was discovered recently that they appear before menarche. It was shown that the microbiota of most girls before menarche was predominantly composed of various *Lactobacillus* species, observed during the early to mid-puberty stages [73].

The vaginal microbiome is known to play a key role in the maintenance of vaginal pH and the prevention of various urogenital diseases. Evidence suggests that vaginal lactobacilli confer a level of protection against bacterial vaginosis, sexually transmitted diseases, and UTIs [74].

## 7. Innate Immunity in the Vagina

Current knowledge and key concepts link the microbiome to the development and function of the immune system [75]. Many studies continue to indicate that a vaginal bacterial community dominated by lactobacilli plays a significant role in driving the innate immune response [76]. The health of both pregnant and non-pregnant women is supported by a higher concentration of vaginal lactobacilli. Their resistance to infections is also enhanced under such conditions [77].

When established in the vaginal epithelium, lactobacilli successfully inhibit other bacteria using several well-known factors such as lowering the pH, thanks to the production of a variety of the organic acids, bacteriocin production, and dissolving biofilm structures [78]. The low pH of vaginal secretions is linked to the lactic acid produced by all *Lactobacillus* species; however, in fact, except for lactic acid, there are multiple organic acids produced by lactobacilli in the vagina at lower amounts that possess much stronger antimicrobial activities. These are citric, succinic, and phenyllactic acid, hydroxyphenyllactic acid, indole lactic acid, 3-phenyllactic acid, 4-hydroxyphenyllactic acid, and 2-hydroxyisocaproic acid [79,80,81].

The innate immune system of the vagina is mainly composed of a tissue barrier, immune cells, and innate immune molecules. The antigen-presenting cells, macrophages, and natural killer (NK) cells in the lower genital tract can use their pattern recognition receptors(such as Toll-like receptors (TLRs), c-type lectin receptors, and NOD receptors) to combine with different bacterial products like *Lactobacillus* products and transmit inflammatory signals in the cell, activating many kinds of T cells and increasing the IgA antibody levels of plasma cells from B cells. Therefore, the antimicrobial acids and peptides produced by vaginal lactobacilli should be considered part of innate immunity in the vagina [82].

Moreover, the microbiota of the vagina, which are vastly predominated by several species of *Lactobacillus sensu lato*, play an important role in the induction of both adaptive and innate memory [83]. Even with intensive sequencing studies, the molecular mechanisms of *Lactobacillus* predominance in the vaginal microbiota remain unexplored. Other elements that play a role in vaginal defense are mannose-binding lectin (MBL), vaginal antimicrobial peptides (AMPs), and immunoglobulins A and G (IgA and IgG).

The presence of protective *Lactobacillus* species in the vagina leads to the reduced activation of vaginal epithelial cells. Numerous epidemiological studies have shown that women with a vaginal microbiota predominantly consisting of *L. crispatus* have lower levels of pro-inflammatory cytokines, such as interleukin (IL)-1α and IL-1β, as well as chemokines like IL-8, interferon-gamma inducible protein (IP-10), and macrophage inflammatory protein (MIP)-3α in their cervicovaginal secretions [84].

## 8. Adaptive Immunity

The female genital tract contains most elements of both the adaptive and innate mucosal immune systems. Unlike other mucosal areas, the vagina has a limited amount of local lymphoid tissue. While other mucosal sites mainly produce IgA and IgM, the predominant immunoglobulin secreted in the vagina and cervix is IgG. Vaginal epithelial cells generate various antimicrobial substances and are equipped with membrane-bound Toll-like receptors that detect pathogen-associated molecular patterns. This detection triggers the production of pro-inflammatory cytokines and antigen-specific immune responses. Additionally, the endocervix and vagina can locally produce IgG and IgA antibodies in response to infections [85,86]. Moreover, as mentioned above, *Lactobacillus sensu lato* plays an important role in the induction of adaptive memory [83].

We need to agree with Vaneechoutte [66] that, despite over a century of research, significant debates persist on fundamental issues, with the ongoing publication of controversial findings. Given the substantial effects of vaginal microbiology on women’s health and pregnancy outcomes, it is crucial to address these controversies. Doing so will enhance our understanding of vaginal microbiology and its relationship with vaginal immunity, leading to improvements in vaginal probiotics and approaches aimed at helping to maintain or restore *Lactobacillus* dominance.

## 9. Conclusions

The name *Lactobacillus* is broadly recognized and utilized by the medical community, the food and health-related industries, and the general public, and is also featured in national and international regulations. Despite being a traditional term that encompasses multiple genera and numerous species, it remains widely accepted. The bacteria have hindered efforts to study the ecology, physiology, evolution, and practical uses of a significant group of organisms. In reality, these bacteria are genetically distinct and exhibit considerable diversity in their metabolism, ecology, and functions. As a result, they can no longer be grouped within the same genus, and thus, a new taxonomy has been proposed for them, and its adoption will stimulate a new wave of research on the relationships between the taxonomic position and characteristics of the bacterial strains formerly placed in the *Lactobacillus* genus.

*Lactobacilli* have colonized multiple areas of the human body such as the digestive tract, including the oral cavity, and the female genital tract. The association between *Lactobacilli* and humans is a mutualistic relationship, with many *Lactobacillus* species offering the host protection from pathogens in return for accommodation and nutrients. Altogether, the *Lactobacillus* name represents the most promising genera described to possess potential beneficial effects for different diseases including inflammatory processes and immunological alterations. These data will undoubtedly yield new insights into the significance of these bacteria for our health, even though many of them will be assigned new names.

## References

[1] Zheng J., Wittouck S., Salvetti E., Franz C.M.A.P., Harris H.M.B., Mattarelli P., O’Toole P.W., Pot B., Vandamme P., Walter J. (2020). A taxonomic note on the genus *Lactobacillus*: Description of 23 novel genera, emended description of the genus *Lactobacillus Beijerinck* 1901, and union of Lactobacillaceae and Leuconostocaceae. Int. J. Syst. Evol. Microbiol..

[2] O’Callaghan J., O’Toole P.W. (2013). *Lactobacillus*: Host-microbe relationships. Curr. Top. Microbiol. Immunol..

[3] De Angelis M., Calasso M., Cavallo N., Di Cagno R., Gobbetti M. (2016). Functional proteomics within the genus *Lactobacillus*. Proteomics.

[4] Seddik H.A., Bendali F., Gancel F., Fliss I., Spano G., Drider D. (2017). *Lactobacillus plantarum* and Its Probiotic and Food Potentialities. Probiotics Antimicrob. Proteins.

[5] Hill C., Sanders M.E. (2013). Rethinking “probiotics”. Gut Microbes.

[6] Idicula D.V., Krishnaprasad N., Parappilly S.J., Joy N., Balan J., George S.M. (2023). Salutary attributes of probiotic human gut lactobacilli for gut health. Lett. Appl. Microbiol..

[7] Thursby E., Juge N. (2017). Introduction to the human gut microbiota. Biochem. J..

[8] Li J., Jia H., Cai X., Zhong H., Feng Q., Sunagawa S., Arumugam M., Kultima J.R., Prifti E., Nielsen T. (2014). An integrated catalog of reference genes in the human gut microbiome. Nat. Biotechnol..

[9] Cavaillon J.M., Legout S. (2016). Centenary of the death of Elie Metchnikoff: A visionary and an outstanding team leader. Microbes Infect..

[10] Hornef M.W., Torow N. (2020). ‘Layered immunity’ and the ‘neonatal window of opportunity’—Timed succession of non-redundant phases to establish mucosal host-microbial homeostasis after birth. Immunology.

[11] Browne H.P., Shao Y., Lawley T.D. (2022). Mother-infant transmission of human microbiota. Curr. Opin. Microbiol..

[12] Bischoff S.C., Barbara G., Buurman W., Ockhuizen T., Schulzke J.D., Serino M., Tilg H., Watson A., Wells J.M. (2014). Intestinal permeability—A new target for disease prevention and therapy. BMC Gastroenterol..

[13] Strus M., Helwich E., Lauterbach R., Rzepecka-Węglarz B., Nowicka K., Wilińska M., Szczapa J., Rudnicka M., Sławska H., Szczepański M. (2018). Effects of oral probiotic supplementation on gut Lactobacillus and Bifidobacterium populations and the clinical status of low-birth-weight preterm neonates: A multicenter randomized, double-blind, placebo-controlled trial. Infect. Drug Resist..

[14] Golińska E., Strus M., Tomusiak-Plebanek A., Więcek G., Kozień Ł., Lauterbach R., Pawlik D., Rzepecka-Węglarz B., Kędzierska J., Dorycka M. (2020). Coagulase-Negative Staphylococci Contained in Gut Microbiota as a Primary Source of Sepsis in Low- and Very Low Birth Weight Neonates. J. Clin. Med..

[15] Wójkowska-Mach J., Chmielarczyk A., Strus M., Lauterbach R., Heczko P. (2019). Neonate Bloodstream Infections in Organization for Economic Cooperation and Development Countries: An Update on Epidemiology and Prevention. J. Clin. Med..

[16] Urushiyama D., Suda W., Ohnishi E., Araki R., Kiyoshima C., Kurakazu M., Sanui A., Yotsumoto F., Murata M., Nabeshima K. (2017). Microbiome profile of the amniotic fluid as a predictive biomarker of perinatal outcome. Sci. Rep..

[17] Satokari R., Grönroos T., Laitinen K., Salminen S., Isolauri E. (2009). Bifidobacterium and Lactobacillus DNA in the human placenta. Lett. Appl. Microbiol..

[18] Rehbinder E.M., Lødrup Carlsen K.C., Staff A.C., Angell I.L., Landrø L., Hilde K., Gaustad P., Rudi K. (2018). Is amniotic fluid of women with uncomplicated term pregnancies free of bacteria?. Am. J. Obstet. Gynecol..

[19] Liu Y., Li X., Zhu B., Zhao H., Ai Q., Tong Y., Qin S., Feng Y., Wang Y., Wang S. (2020). Midtrimester amniotic fluid from healthy pregnancies has no microorganisms using multiple methods of microbiologic inquiry. Am. J. Obstet. Gynecol..

[20] Bagg J., MacFarlane T.W., Poxton I.R., Smith A.J. (2006). Essentials of Microbiology for Dental Students.

[21] Hegde S., Munshi A.K. (1998). Influence of the maternal vaginal microbiota on the oral microbiota of the newborn. J. Clin. Pediatr. Dent..

[22] Mändar R., Mikelsaar M. (1996). Transmission of mother’s microflora to the newborn at birth. Biol. Neonate.

[23] He Q., Kwok L.Y., Xi X., Zhong Z., Ma T., Xu H., Meng H., Zhao F., Zhang H. (2020). The meconium microbiota shares more features with the amniotic fluid microbiota than the maternal fecal and vaginal microbiota. Gut Microbes.

[24] Bäckhed F., Roswall J., Peng Y., Feng Q., Jia H., Kovatcheva-Datchary P., Li Y., Xia Y., Xie H., Zhong H. (2015). Dynamics and Stabilization of the Human Gut Microbiome during the First Year of Life. Cell Host Microbe.

[25] Soto A., Martín V., Jiménez E., Mader I., Rodríguez J.M., Fernández L. (2014). Lactobacilli and bifidobacteria in human breast milk: Influence of antibiotherapy and other host and clinical factors. J. Pediatr. Gastroenterol. Nutr..

[26] Rodríguez J.M. (2014). The origin of human milk bacteria: Is there a bacterial entero-mammary pathway during late pregnancy and lactation?. Adv. Nutr..

[27] Lyons K.E., Ryan C.A., Dempsey E.M., Ross R.P., Stanton C. (2020). Breast Milk, a Source of Beneficial Microbes and Associated Benefits for Infant Health. Nutrients.

[28] Dominguez-Bello M.G., Costello E.K., Contreras M., Magris M., Hidalgo G., Fierer N., Knight R. (2010). Delivery mode shapes the acquisition and structure of the initial microbiota across multiple body habitats in newborns. Proc. Natl. Acad. Sci. USA.

[29] Rinninella E., Raoul P., Cintoni M., Franceschi F., Miggiano G.A.D., Gasbarrini A., Mele M.C. (2019). What is the Healthy Gut Microbiota Composition? A Changing Ecosystem across Age, Environment, Diet, and Diseases. Microorganisms.

[30] Azad M.A.K., Sarker M., Li T., Yin J. (2018). Probiotic Species in the Modulation of Gut Microbiota: An Overview. Biomed. Res. Int..

[31] Gupta V., Garg R. (2009). Probiotics. Indian J. Med. Microbiol..

[32] Kim S., Jazwinski S.M. (2018). The Gut Microbiota and Healthy Aging: A Mini-Review. Gerontology.

[33] Mármol I., Sánchez-de-Diego C., Pradilla Dieste A., Cerrada E., Rodriguez Yoldi M.J. (2017). Colorectal Carcinoma: A General Overview and Future Perspectives in Colorectal Cancer. Int. J. Mol. Sci..

[34] Troost F.J., van Baarlen P., Lindsey P., Kodde A., de Vos W.M., Kleerebezem M., Brummer R.J. (2008). Identification of the transcriptional response of human intestinal mucosa to Lactobacillus plantarum WCFS1 in vivo. BMC Genom..

[35] van Baarlen P., Troost F.J., van Hemert S., van der Meer C., de Vos W.M., de Groot P.J., Hooiveld G.J., Brummer R.J., Kleerebezem M. (2009). Differential NF-kappaB pathways induction by *Lactobacillus plantarum* in the duodenum of healthy humans correlating with immune tolerance. Proc. Natl. Acad. Sci. USA.

[36] Liu Y., Tian X., He B., Hoang T.K., Taylor C.M., Blanchard E., Freeborn J., Park S., Luo M., Couturier J. (2019). *Lactobacillus reuteri* DSM 17938 feeding of healthy newborn mice regulates immune responses while modulating gut microbiota and boosting beneficial metabolites. Am. J. Physiol. Gastrointest. Liver Physiol..

[37] Allaire J.M., Crowley S.M., Law H.T., Chang S.Y., Ko H.J., Vallance B.A. (2018). The Intestinal Epithelium: Central Coordinator of Mucosal Immunity. Trends Immunol..

[38] Bentley R., Meganathan R. (1982). Biosynthesis of vitamin K (menaquinone) in bacteria. Microbiol. Rev..

[39] Plaza S.M. (2003). The anticancer effects of vitamin K. Altern. Med. Rev..

[40] Rook G.A., Lowry C.A., Raison C.L. (2015). Hygiene and other early childhood influences on the subsequent function of the immune system. Brain Res..

[41] Newburg D.S. (2005). Innate immunity and human milk. J. Nutr..

[42] Cheng C., Zhang L., Mu J., Tian Q., Liu Y., Ma X., Fu Y., Liu Z., Li Z. (2021). Effect of *Lactobacillus johnsonii* Strain SQ0048 on the TLRs-MyD88/NF-κB Signaling Pathway in Bovine Vaginal Epithelial Cells. Front. Vet. Sci..

[43] Jiang Y., Lü X., Man C., Han L., Shan Y., Qu X., Liu Y., Yang S., Xue Y., Zhang Y. (2012). *Lactobacillus acidophilus* induces cytokine and chemokine production via NF-κB and p38 mitogen-activated protein kinase signaling pathways in intestinal epithelial cells. Clin. Vaccine Immunol..

[44] Kim Y.G., Ohta T., Takahashi T., Kushiro A., Nomoto K., Yokokura T., Okada N., Danbara H. (2006). Probiotic *Lactobacillus casei* activates innate immunity via NF-kappaB and p38 MAP kinase signaling pathways. Microbes Infect..

[45] Shin D., Chang S.Y., Bogere P., Won K., Choi J.Y., Choi Y.J., Lee H.K., Hur J., Park B.Y., Kim Y. (2019). Beneficial roles of probiotics on the modulation of gut microbiota and immune response in pigs. PLoS ONE..

[46] Richards P.J., Flaujac Lafontaine G.M., Connerton P.L., Liang L., Asiani K., Fish N.M., Connerton I.F. (2020). Galacto-Oligosaccharides Modulate the Juvenile Gut Microbiome and Innate Immunity To Improve Broiler Chicken Performance. mSystems.

[47] Forsythe P., Bienenstock J. (2010). Immunomodulation by commensal and probiotic bacteria. Immunol. Investig..

[48] Rocha-Ramírez L.M., Pérez-Solano R.A., Castañón-Alonso S.L., Moreno Guerrero S.S., Ramírez Pacheco A., García Garibay M., Eslava C. (2017). Probiotic Lactobacillus Strains Stimulate the Inflammatory Response and Activate Human Macrophages. J. Immunol. Res..

[49] Marcinkiewicz J., Ciszek M., Bobek M., Strus M., Heczko P.B., Kurnyta M., Biedroń R., Chmielarczyk A. (2007). Differential inflammatory mediator response in vitro from murine macrophages to lactobacilli and pathogenic intestinal bacteria. Int. J. Exp. Pathol..

[50] Jakubczyk D., Leszczyńska K., Górska S. (2020). The Effectiveness of Probiotics in the Treatment of Inflammatory Bowel Disease (IBD)-A Critical Review. Nutrients.

[51] Ni J., Wu G.D., Albenberg L., Tomov V.T. (2017). Gut microbiota and IBD: Causation or correlation?. Nat. Rev. Gastroenterol. Hepatol..

[52] Strus M., Brzychczy-Włoch M., Kochan P., Heczko P. (2004). Nadtlenek wodoru, wytwarzany przez bakterie z rodzaju *Lactobacillus*, jako czynnik regulujacy mikroflore pochwy [Hydrogen peroxide produced by *Lactobacillus* species as a regulatory molecule for vaginal microflora]. Med. Dosw. Mikrobiol..

[53] Tomusiak-Plebanek A., Heczko P., Skowron B., Baranowska A., Okoń K., Thor P.J., Strus M. (2018). Lactobacilli with superoxide dismutase-like or catalase activity are more effective in alleviating inflammation in an inflammatory bowel disease mouse model. Drug Des. Dev. Ther..

[54] Gevers D., Kugathasan S., Denson L.A., Vázquez-Baeza Y., Van Treuren W., Ren B., Schwager E., Knights D., Song S.J., Yassour M. (2014). The treatment-naive microbiome in new-onset Crohn’s disease. Cell Host Microbe.

[55] Fyderek K., Strus M., Kowalska-Duplaga K., Gosiewski T., Wedrychowicz A., Jedynak-Wasowicz U., Sładek M., Pieczarkowski S., Adamski P., Kochan P. (2009). Mucosal bacterial microflora and mucus layer thickness in adolescents with inflammatory bowel disease. World J. Gastroenterol..

[56] Pilarczyk-Zurek M., Chmielarczyk A., Gosiewski T., Tomusiak A., Adamski P., Zwolinska-Wcislo M., Mach T., Heczko P.B., Strus M. (2013). Possible role of *Escherichia coli* in propagation and perpetuation of chronic inflammation in ulcerative colitis. BMC Gastroenterol..

[57] Pilarczyk-Zurek M., Strus M., Adamski P., Heczko P.B. (2016). The dual role of *Escherichia coli* in the course of ulcerative colitis. BMC Gastroenterol..

[58] Liu Y., Fatheree N.Y., Mangalat N., Rhoads J.M. (2012). *Lactobacillus reuteri* strains reduce incidence and severity of experimental necrotizing enterocolitis via modulation of TLR4 and NF-κB signaling in the intestine. Am. J. Physiol. Gastrointest. Liver Physiol..

[59] Munyaka P.M., Khafipour E., Ghia J.E. (2014). External influence of early childhood establishment of gut microbiota and subsequent health implications. Front. Pediatr..

[60] Ashraf R., Shah N.P. (2014). Immune system stimulation by probiotic microorganisms. Crit. Rev. Food Sci. Nutr..

[61] Amabebe E., Anumba D.O.C. (2018). The Vaginal Microenvironment: The Physiologic Role of Lactobacilli. Front. Med..

[62] Onderdonk A.B., Delaney M.L., Fichorova R.N. (2016). The Human Microbiome during Bacterial Vaginosis. Clin. Microbiol. Rev..

[63] Muzny C.A., Blanchard E., Taylor C.M., Aaron K.J., Talluri R., Griswold M.E., Redden D.T., Luo M., Welsh D.A., Van Der Pol W.J. (2018). Identification of Key Bacteria Involved in the Induction of Incident Bacterial Vaginosis: A Prospective Study. J. Infect. Dis..

[64] Hasan Z., Netherland M., Hasan N.A., Begum N., Yasmin M., Ahmed S. (2024). An insight into the vaginal microbiome of infertile women in Bangladesh using metagenomic approach. Front. Cell. Infect. Microbiol..

[65] Ravel J., Gajer P., Abdo Z., Schneider G.M., Koenig S.S.K., McCulle S.L., Karlebach S., Gorle R., Russell J., Tacket C.O. (2011). Vaginal microbiome of reproductive-age women. Proc. Natl. Acad. Sci. USA.

[66] Vaneechoutte M. (2017). The human vaginal microbial community. Res. Microbiol..

[67] El Aila N.A., Tency I., Claeys G., Verstraelen H., Saerens B., Santiago G.L., De Backer E., Cools P., Temmerman M., Verhelst R. (2009). Identification and genotyping of bacteria from paired vaginal and rectal samples from pregnant women indicates similarity between vaginal and rectal microflora. BMC Infect. Dis..

[68] Gustafsson R.J., Ahrné S., Jeppsson B., Benoni C., Olsson C., Stjernquist M., Ohlsson B. (2011). The *Lactobacillus flora* in vagina and rectum of fertile and postmenopausal healthy Swedish women. BMC Womens Health.

[69] Reid G., Charbonneau D., Erb J., Kochanowski B., Beuerman D., Poehner R., Bruce A.W. (2003). Oral use of *Lactobacillus rhamnosus* GR-1 and *L. fermentum* RC-14 significantly alters vaginal flora: Randomized, placebo-controlled trial in 64 healthy women. FEMS Immunol. Med. Microbiol..

[70] Heczko P.B., Tomusiak A., Adamski P., Jakimiuk A.J., Stefański G., Mikołajczyk-Cichońska A., Suda-Szczurek M., Strus M. (2015). Supplementation of standard antibiotic therapy with oral probiotics for bacterial vaginosis and aerobic vaginitis: A randomised, double-blind, placebo-controlled trial. BMC Womens Health.

[71] Koirala R., Gargari G., Arioli S., Taverniti V., Fiore W., Grossi E., Anelli G.M., Cetin I., Guglielmetti S. (2020). Effect of oral consumption of capsules containing *Lactobacillus paracasei* LPC-S01 on the vaginal microbiota of healthy adult women: A randomized, placebo-controlled, double-blind crossover study. FEMS Microbiol. Ecol..

[72] Witkin S.S., Linhares I.M. (2017). Why do lactobacilli dominate the human vaginal microbiota?. BJOG.

[73] Hickey R.J., Zhou X., Settles M.L., Erb J., Malone K., Hansmann M.A., Shew M.L., Van Der Pol B., Fortenberry J.D., Forney L.J. (2015). Vaginal microbiota of adolescent girls prior to the onset of menarche resemble those of reproductive-age women. mBio..

[74] Neugent M.L., Hulyalkar N.V., Nguyen V.H., Zimmern P.E., De Nisco N.J. (2020). Advances in Understanding the Human Urinary Microbiome and Its Potential Role in Urinary Tract Infection. mBio.

[75] Zheng D., Liwinski T., Elinav E. (2020). Interactions between microbiota and immunity in health and disease. Cell Res..

[76] Smith S.B., Ravel J. (2017). The vaginal microbiota, host defence and reproductive physiology. J. Physiol..

[77] Witkin S.S., Linhares I.M., Giraldo P. (2007). Bacterial flora of the female genital tract: Function and immune regulation. Best. Pract. Res. Clin. Obstet. Gynaecol..

[78] Saunders S., Bocking A., Challis J., Reid G. (2007). Effect of Lactobacillus challenge on Gardnerella vaginalis biofilms. Colloids Surf. B Biointerfaces.

[79] Axel C., Brosnan B., Zannini E., Peyer L.C., Furey A., Coffey A., Arendt E.K. (2016). Antifungal activities of three different *Lactobacillus* species and their production of antifungal carboxylic acids in wheat sourdough. Appl. Microbiol. Biotechnol..

[80] Szczerbiec D., Piechocka J., Głowacki R., Torzewska A. (2022). Organic Acids Secreted by *Lactobacillus* spp. Isolated from Urine and Their Antimicrobial Activity against Uropathogenic Proteus mirabilis. Molecules.

[81] Qian Z., Zhu H., Zhao D., Yang P., Gao F., Lu C., Yin Y., Kan S., Chen D. (2021). Probiotic *Lactobacillus* sp. Strains Inhibit Growth, Adhesion, Biofilm Formation, and Gene Expression of Bacterial Vaginosis-Inducing Gardnerella vaginalis. Microorganisms.

[82] Mei C., Yang W., Wei X., Wu K., Huang D. (2019). The Unique Microbiome and Innate Immunity During Pregnancy. Front. Immunol..

[83] McCoy K.D., Burkhard R., Geuking M.B. (2019). The microbiome and immune memory formation. Immunol. Cell Biol..

[84] Nunn K.L., Forney L.J. (2016). Unraveling the dynamics of the human vaginal microbiome. Yale J. Biol. Med..

[85] Masson L., Barnabas S., Deese J., Lennard K., Dabee S., Gamieldien H., Jaumdally S.Z., Williamson A.L., Little F., Van Damme L. (2019). Inflammatory cytokine biomarkers of asymptomatic sexually transmitted infections and vaginal dysbiosis: A multicentre validation study. Sex. Transm. Infect..

[86] Naz R.K. (2012). Female genital tract immunity: Distinct immunological challenges for vaccine development. J. Reprod. Immunol..

